# Moving from the Mainstream to the Margins: Lessons in Culture and Power

**DOI:** 10.1007/s10896-018-9984-1

**Published:** 2018-08-31

**Authors:** Ruby White Starr

**Affiliations:** Latinos United for Peace and Equity, Caminar Latino, P.O. Box 48623, Doraville, GA 30362 USA

**Keywords:** Domestic violence, Culture, Power, Oppression, Underserved, Culturally-specific communities, Meaningful collaborations

## Abstract

This article highlights the ways in which power is conceptualized, activated, and institutionalized in American culture. Drawing from research and the author’s experience within mainstream and culturally-specific organizations in the violence against women field, this article exposes the subtle, yet pervasive mechanisms that lead to the marginalization of culturally specific communities and smaller, typically culturally specific, community-based organizations. By design and unconsciously, researchers, mainstream organization, and leaders often perpetuate a system designed to localize research, evaluation, services and resources for white people, organizations and institutions. This occurs for example, when researchers center and elevate a “gold-standard” of evidence-based practices, research, and evaluation that share no frame of reference to those being “studied” and most effected. It also happens when organizations marginalize culturally specific community members and organizations by seeking their participation at the final stages rather than at the conception of projects. The author provides concrete recommendations that researchers, providers, and leaders can adopt to counteract institutional oppression and help move culturally-specific communities and organizations from the margins to the center.

When differences are identified between the mainstream^1^ and those labeled as “others,” they are called cultural. I have come to understand, however, that these differences are more often a reflection of power. Power is the ability to decide what a problem is, to decide what needs to be done about it, to decide who will be included to solve it, in what capacity, and with what resources. Power is maintained by possessing multiple attributes of the dominant culture, which in America includes “whiteness,” speaking English as a primary language, and being male, Christian, physically able, economic resourced, and heterosexual. These attributes create the norms against which all other sub groups are compared and judged. Power includes not having to recognize one’s culture^2^ as the norm or to acknowledge one’s access to resources, connections, and status. As organisms of the dominant culture, none of us are immune. Even as a Latina woman, I have both confronted and contributed to this over the last 25 years working in the violence against women field. This commentary will describe those experiences and share lessons on what can be done differently to promote racial equity.

I began my career in the early 90’s at the Committee to Aid Abused Women (CAAW); a mainstream, feminist, grassroots, advocacy organization founded in Reno, Nevada by Joni Kaiser in 1977. By the year 2000, I worked at the National Council of Juvenile and Family Court Judges founded in 1937 by a group of judges seeking to improve the effectiveness of the nation’s juvenile courts. For more than two decades within these structures, despite being a woman of color, I was part of the mainstream that worked to “integrate” cultural relevancy, instead of unweaving the fabric of oppression that blankets us all. This structural racism was not something that I, or that some people or institutions, chose to practice. Structural racism in the U.S. is the normalization and legitimatization of an array of dynamics (historical, cultural, institutional, and interpersonal) that routinely advantage whites while producing cumulative and chronic adverse outcomes for people of color (Lawrence and Keleher 2004). It is a system of hierarchy and inequity, primarily characterized by white supremacy.^3^ It is a feature of the social, economic and political systems in which we all exist; where research, public policies, institutional practices, cultural representations, and other norms work in various and reinforcing ways to perpetuate racial group inequity. In theory, this is not groundbreaking information, but in practice, I did not fully comprehend its significance until it evolved into my lived reality.

That evolution gained momentum in 2004 when I became a member of the Board of Directors for the National Latino Alliance for the Elimination of Domestic Violence (Alianza), the most prominent national Latino domestic violence organization of its time. The organization’s mission was to promote understanding, initiate and sustain dialogue, and generate solutions that move toward the elimination of domestic violence affecting Latino communities. I became President of the Alianza board within a year and served in that capacity until 2012. During those years, few, if any, mainstream organizations (other than those which board members had existing affiliations) reached out to collaborate. On the contrary, criticism and barriers mounted when theories or strategies veered too far away from mainstream feminist models. For example, Dr. Julia Perilla, a prominent Latina leader, Alianza board member (at that time), and faculty member in the Department of Psychology at Georgia State University, faced years of criticism for her culturally responsive practice and research approaches. Dr. Perilla conducted domestic violence education groups for women, children and men separately but in the same building simultaneously. Despite it being what the Latina women in her programs requested, practitioners at mainstream programs, who in one breath advocated that providers listen and respond to what survivors say they want, then criticized it as an unsafe practice. In the view of mainstream practitioners, safety was characterized by leaving the relationship and accepting that “batterers” can’t change. These mainstream practitioners failed to consider that many women lived and intended to remain with their partner, drove to and attended group while their partner waited outside because of transportation limitations, or had partners with complicated histories of their own abuse and oppression.

In June of 2015, I was recruited by Casa de Esperanza to lead their national efforts which operated as the National Latin@ Network for Healthy Families and Communities (NLN). I remained at the NLN until March of 2018 when I joined Caminar Latino to launch our own national project – Latinos United for Peace and Equity to foster more liberative systems and approches for those impacted by violence. At the NLN, I had more power within my own organization than in previous positions in the mainstream, but, like Alianza, it was juxtaposed to power of a lesser degree within the broader domestic violence field because we worked at the intersections. This was evident in the way we were included or excluded in the work. For example, when I was in the mainstream, a request to partner developed from relationships that were built doing common work. We sat at, and in many cases set, numerous tables to explore issues, identify problems, name experts, and deliberate solutions. When resources were available, we discussed approaches, delineated responsibilities, allocated resources, and entered into subcontracts. When agencies reached out, they expected to pay for a service unless it was part of our current funding structure. I brought knowledge of this “power” to my new position, but not always its access.

At the NLN, requests to collaborate came from organizations that had never, or rarely, reached out to us until a funding proposal required “cultural responsiveness.” These requests occurred after the project need, scope, and approach was decided and without information as to what that was because of “tight turnarounds.” Not only did many requests to “partner” not include compensation, but they were met with disappointment that our “very important issues would not be represented” when we could not participate at our own expense. As the NLN, we were more resourced than most culturally-specific organizations, but being federally-funded, it came with many restrictions. When requests did include compensation, it was not for a subcontract that allowed for staffing, equipment, supplies, overhead, etc., but instead for individual staff to serve as consultants with a daily rate. To keep the consulting agreement as low cost as possible, these requests often involved just a few days to review the materials that the organization and their mainstream partners developed after several discussions and meetings we would not be present at or to “integrate” culture into a final tool or product.

Each of these examples is just a microcosm that revealed not only a difference in culture, but a difference in power. The cyclical result is that culturally specific,^4^ community-based organizations are undervalued, overburdened, and unable to climb out of the margins. For example, when information is not provided in Spanish, culturally specific, community-based organizations may translate it, so their constituencies can have access. As a result, they continue to be contacted and expected to translate materials and surveys for free by much more heavily resourced organizations and institutions. Culturally specific, community-based organizational staff will often review materials, serve on task force’s, and “partner” on projects, despite being brought in late in the process and without funding, to expose barriers and problem solve because they are committed to address the issues. However, since the need is so great, they are often stretched beyond their capacity which can lead to the appearance that they lack competence, are unreliable, or not fully invested. Due to the treatment of culture as a secondary issue (if at all), culturally specific, community-based organizations are also not afforded the opportunity to build their capacity as part of a process, they remain unaware of the unwritten rules designed and navigated by mainstream organizations, and their insights and viewpoints are dismissed as ancillary or absent of “the big picture.” I came to recognize that the more fitting term for the collaborations that mainstream organizations had with culturally specific organizations is **knowledge appropriation**. Knowledge appropriation occurs when the dominant culture adopts elements of a culturally specific community without an equitable cultural exchange to determine (with those communities) how to acquire, interpret, use, and distribute that knowledge.

None of us is immune. I am a woman of color, operated a domestic violence shelter, led programs in a judicial organization, sat on culturally-specific projects and advisory councils across the country, and secured, implemented and directed more than 50 federal initiatives over the course of my career. Just like my complicity was not a choice, it was a byproduct, my understanding is not innate, it is evolved. Although we cannot be vaccinated from participating in systems and structures that oppress, what we can do to create change is facilitate a more deliberate sharing of power. Not the kind of power that lives in a soundbite. We need the kind of power that begins with a commitment to understand and resist structural racism as an **explicit** part of our work and does not end there.

To understand structural racism, researchers, policy makers and providers need to recognize it, analyze how each issue we work on is shaped by it, admit how what we design is influenced by it, and acknowledge how each of us, our work, and our organizations is shaped by it. To resist structural racism, researchers, policy makers and providers need to acknowledge our complicity in perpetuating it, cease amassing power, and instead engage in collective problem-solving and decision-making, equitable partnerships, and meaningful collaboration. To better appreciate what this means in practice, and for the research field, I offer the following suggestions and reflections to share power and promote racial equity: recognize American culture, draw parallels between sexism and racial oppression, use a multilayered approach right from the start, and engage in meaningful collaboration. The following sections will explore these recommendations in depth.

## Recognize American Culture

All meaning is assigned. We interpret other people’s behavior through our own cultural filter of what our worldview tells us is happening. Despite this, requests for training and technical assistance I’ve received in 20 years of providing race and anti-oppression assistance, almost exclusively, involve inquiries for information to understand other cultures. Typically, when service providers, policy makers, or researchers seek “culturally competence,” they are attempting to serve, govern, or evaluate those with which they share no frame of reference. In cases such as these, I cannot stress enough the importance to first, know thyself. For example, recognize the tools you use to measure success and failure. Unpack how those tools were created and by whom. For instance, when a child serves as a caregiver to a parent or sibling, is it a pathology; destructive parentification? Or is it, as some communities of color believe, a positive resilience factor that can promote family unity and bonding? This is determined by culture.

No one is culture free. America and Americans have a culture. No one American is quite like any other American, but a handful of core values and beliefs underlie and permeate the national culture. These values and beliefs do not apply across the board in every situation, and in many instances, Americans may even act in ways that directly contradict them (as is the case with other cultures), but they are still at the heart of Americas cultural ethos. These values and beliefs include principles of white supremacy that are not only socially accepted, but socially enforced to maintain societal control. For example, “good intentions are good enough.” This principle is illustrated in case after case where a person of color draws attention to cultural incapacity and blindness or racism and met with the response of “that wasn’t my/our intent”—an awareness gap that elevates one’s intention at the expense of the impact of the event. Other socially acceptable and reinforced values in America include: a right to comfort, paternalism, either/or thinking, progress means more and bigger and better, strict adherence to time, power hoarding, fear of open conflict, and individualism.^5^ Each of these, and other constructs merit their own discussion as they infuse bias into research, policy, and practice, but cannot be adequately addressed here given the restricted page limits.

American values such as objectivity, exclusivity, and “worship of the written word” are especially problematic for knowledge production, evaluation, and research. Calls for evidence-based practice and discrediting of what has not been documented by a “credible” source, derive from world views in America that there is a “knower” and that which can and must be known. The American Evaluation Association Statement on Cultural Competence in Evaluation states that “evaluations cannot be culture free. Those who engage in evaluation do so from perspectives that reflect their values, their ways of viewing the world, and their culture. Culture shapes the ways in which evaluation questions are conceptualized, which in turn influence what data are collected, how the data will be collected and analyzed, and how data are interpreted” (American Evaluation Association 2011). In fact, in many minority and indigenous communities there is a history of inappropriate use of research or evaluation in ways that violated basic human rights (American Evaluation Association 2011). Further, not all cultures or world views ascribe to belief that there is a “knower,” and that or those which can be “known.”

According to Thomas (2017), concepts and terms such as “objectivity,” “scientific,” “valid,” “reliable,” and “rationality” prove to be extremely powerful in academia and scholarship, yet the great irony of that power is that these concepts and terms are veneer for maintaining white male power ― inequity grounded in the racism and sexism that academics are prone to refute in their rhetoric while maintaining in their practices. As Fine (2010) describes, “the language of randomized clinical trials and experimental designs dominate the evaluation field. Represented as the gold standard of validity, these designs equate distance with objectivity, local context as a variable to be controlled and individual-level quantifiable outcomes as the primary form of evidence. Participatory evaluations on structural racism challenge these assumptions theoretically and scientifically.”

To share power and promote racial equity one should:

- Utilize and elevate participatory action research and include participants to interpret and present results. (See for example: Serrata et al. 2017)
- Recognize the limits of knowledge production and challenge historical assumptions regarding professionalization and “objectivity.”
- Investigate the historical misuse of research and evaluation for communities of color and other marginalized groups.
- Avoid asking people from culturally-specific communities to justify and “cite” their expert opinions based on years of lived experience and over-relying on them to speak for their “culture.” Credit them for their contributions, whether verbal or in written form.
- Tell stories about racism and assist one another to release the intense feelings that underlie these stories. These stories can include experiences with racism, the racist lies we were told, the times we acted out racism, and the racist attitudes that were held by people around us, as well as the successes we’ve had in fighting racism. Stories are powerful data that is often minimized in research. Actively work to counteract the academic tendency to diminish the power of narrative.

## Draw Parallels between Sexism and Racial Oppression

The first summit on The United State of Women took place on June 14, 2016. During the event, a prominent, congresswomen recalled her early years trying to create change in Congress. She described an instance where white, more senior men in Congress told the female members to “just tell us what you want, and we’ll do it” but the women in Congress demanded to speak and do for themselves. As the event center erupted with excitement, I turned to my colleagues who sat quietly to say, “that’s what white people say to us, tell us what should happen and we’ll do it.”

It’s likely not a popular opinion, but I have found that individuals with a sophisticated understanding and ability to call out sexism, often fail to see how they perpetuate racial oppression. For example, during a policy meeting of a leading task force, a colleague and I took issue with the way a topic, with many cultural implications, was being addressed. That evening, I received an email from a veteran member of the task force who was white. She began that since we had only recently joined the group (which was inaccurate since though I’d been mostly silent, I’d been involved for years), we likely needed help and stated, “so let me explain to you how it works here.” She proceeded to explain how much time a particular group member (also white) had spent thinking about the issue, and directed us to praise various elements of what is presented in the future before contributing anything critical. In another instance, a white feminist researcher applying for a grant to study culturally specific interventions in domestic violence, declined requests to partner from a researcher of color due to a “small ceiling amount,” even though the white researcher did not have other culturally specific partners on the project.

To draw parallels does not mean to make comparisons. To make comparisons, one looks at two (or more) things to see what is different and the same or to decide which is better/worse or more/less important. Inappropriate comparisons include, “I understand racism because I experience sexism, which is just as bad,” or “that happens to others people too,” when members of a marginalized groups draw attention to a disparity such not being represented in research, being over-represented in social control systems, or having their ideas and practices dismissed as not credible due to lack of “evidence.” To draw a parallel, one instead explores how two (or more) distinct things may help explain a concept or relationship. For instance, if one believes it’s inappropriate for a men’s group to call a domestic violence organization to learn how to serve women so they can open a shelter, then one can draw a parallel that asking a culturally specific community to teach you how to “outreach” to them might be inappropriate as well since engagement or partnership would be more appropriate.

To share power and promote racial equity one should:

- Question frames of reference that elevate sexism as a primary issue and others, including racism, as auxiliary.
- Recognize that paternalism is practiced when one expects unearned praise and trust or limits a person or groups’ liberty or autonomy in the name of promoting their own good.
- Write a reflexivity statement^6^ on your views of anti-sexist research, collaboration, and practice expectations and identify how those would relate to a culturally specific context.
- Study feminist work by women of color and allow those frameworks to shape and define research instead of simply “acknowledging” those frameworks as “other” models.
- Allow for different communication styles, attitudes toward conflict, and approaches to completing tasks.
- Do not rationalize limited resources as an excuse to exclude appropriate partners.

## Use a Multilayered Approach Right from the Start

Research, policy and practice that strive to dismantle racism need to account for the most marginalized from their very inception, not incorporate their needs as time and resources allow. Research, policy and practice should explicitly address disparate outcomes based on race and provide mechanisms to reduce those disparities from the start. Cultural adaptations in research, evaluations tested on numerous groups of white people and then one small subset of a culturally specific group, and declarations that “our services are for everyone” are inadequate.

Universal goals are important, but different groups of people need different supports to reach that shared goal and all have different needs. For example, a universal goal might be that all children have access to a pleasurable and meaningful education. But if your strategies evolve from that, you may focus on resources and supports to cultivate, retain, and nurture good teachers and administrators, specialized curricula, and increasing educational standards. These strategies might help middle class children of all races. But if you want to reach low socio-economic status children, you need to add nutritious meals, stable housing, and access to health care to the list of core strategies. If you want to reach African American and Latino children, the list needs to include curricula and pedagogical approaches that teach relevant history and counter the unconscious impact of pervasive negative stereotypes. If you want to bring recent immigrant and limited English proficiency children out of the margins, your strategies would include English language supports, First language supports, and interpretation and outreach in parent’s first language (adapted from: Hinson and Weisenberg 2015). This process would continue to the most marginalized and be the starting place for targeting your approaches.

To share power and promote racial equity one should:

- Deepen your understanding of the intersections of oppression, privilege, and liberation.
- Recognize how racism and oppression impacts the issues you work on, underscore this impact in your tools, projects, and publications, and consider materials ineffectual if they do not explicitly account for the intersection of oppression.
- Take the lead from and partner with culturally-specific technical assistance providers and communities that operate from an anti-racist foundation.
- Put important issues on the table, frame issues around the most marginalized, and put them to work in different situations when examining a problem, designing and implementing research and evaluation, considering policy, or developing interventions.

## Engage in Meaningful Collaboration

Mainstream advocates, system players, and researchers often purport to engage in collaborations with culturally specific organizations that upon further examination, amount to little more than a referral arrangement, language service, or securement of survey or focus group participant assistance. Though these activities are important, they do not produce the kind of relationships and impact that would demonstrate deliberate power-sharing or promote racial equity. Veritably, it is not uncommon for individuals designing and implementing domestic violence intervention models, approaches, and research to frame the issue, decide what problem they will solve, develop logic models, and determine the tools they will develop to respond without ever seeking congruence between these and those that will be most affected—culturally specific communities. Unfortunately, in many cases where a program advocate or researcher does request feedback on activities from consumers, they have not accounted for the compensation of this expertise; expecting consumers to provide their expertise for free.

Frequently, researchers, service providers, and policy makers can fall in to the practice of Trickle-Down Community Engagement. Trickle-down Community Engagement is used to describe the lack of effectively engaging diverse communities in intentional, fully equitable and reciprocal partnerships. It happens as Vu Le (2015) states “when we bypass the people who are most affected by issues, engage and fund larger organizations to tackle these issues, and hope that the people most affected will help out in the effort with little or no resources*”.* Trickle-Down Research and Evaluation can occur with similar dynamics; when mainstream organizations and institutions receive funding to study populations and issues that they share no frame of reference with or to, generalize their values and assumptions to apply to all communities because they are “evidence-based,” adapt mainstream measures and tools created for other purposes, and hope that people most effected, with relevant experience and information will participate at little or no cost.

To engage in meaningful collaborations, projects and initiatives need to effectively engage diverse communities through intentional, fully equitable, and reciprocal partnerships. To avoid Trickle-Down Community Engagement/Research and Evaluation, share power, and promote racial equity one should:

- Review projects and budgets for an equitable distribution of resources. For example, do budgets include resources for culturally specific partners to familiarize themselves and participate in all project activities or are they relegated to a specific task or workgroup? Are there opportunities for learning and capacity building? Can they cover overhead, equipment, office supplies, administrative support, etc.?
- Examine the role and the dynamics you play to perpetuate trickle down. For example, when do you approach partners and in what way? Is the majority of project funding maintained by your organization or research group as the lead to cover program staff for coordination of the project despite significant core expertise living elsewhere? What degree of involvement in priority setting, decision-making, and recognition will partners receive?
- Do not speak for or about marginalized communities without their full input, participation, and consent. Instead identify the culturally specific community-based nonprofits that should tackle the issues.
- Mentor culturally-specific organizations and community members through strategic partnerships when they don’t have the capacity to respond (often due structural racism itself). This includes offering resources to assist you to conceptualize, draft, and submit proposals instead of expecting they use unrestricted program resources (if those exist) or building their capacity to meet the administrative demands of grants management instead of limiting their participation because of the lack of capacity.
- Continually assess how people of color, people who are deaf or have a disability, LGBTQ, and other culturally specific staff and consumers are doing in your institutions. Do they occupy leadership positions? Do they view you as a resource or a system of restrictions and control? Are your approaches closely aligned with what your community reflects? Are your culturally specific partners adequately resourced to participate fully and design strategies from the inception of an idea to implementation?

Meaningful collaborations can promote equity, inclusion, and meaningful engagement.

They will produce more useful tools, minimize tokenism and the replication of institutional oppressions, and contribute to environments where we can share power and foster racial equity.

## Conclusion

The recommendations outlined in this article to recognize American culture, draw parallels between sexism and racial oppression, use a multi-layered approach right from the start, and engage in meaningful collaboration will not end structural racism. The reflections, suggestions, and examples instead can only serve as a primer to help one increase awareness of blind spots, stimulate “aha” moments, inspire further study, and possibly prompt small changes in the way one conceptualizes culture and activates power. In this way we can create more fundamental change for those who need it most; every one of us.

## Footnotes

1 The author uses mainstream to refer to researchers, service providers, organizations, systems, etc. that represent the prevailing or dominant values and practices of a society with little or no focus to operate from the worldview of culturally-specific communities.

2 Culture refers to shared experiences or commonalities that have developed and continue to evolve in relation to changing social and political contexts, based on: race, ethnicity, national origin, sexuality, gender, religion, age, class, language, disability status, immigration status, education, geographic location, or other axes of identification (Warrier 2005).

3 White supremacy is the preferential treatment, privilege and power for white people at the expense of Black, Latino, Asian, Pacific Islander, Native American, Arab and other racially oppressed people.

4 The author uses culturally-specific to refer to services and organizations created by and for specific cultural communities, such as Latino, African American, Asian, Native, Indigenous, Muslim, lesbian, gay, bisexual, transgender, questions (LGBTQ), people who are deaf, people with a disability, immigrants, or other cultural groups with evolving and intersecting identities.

5 See for example: White Supremacy Culture, From Dismantling Racism: A Workbook for Social Change Groups, by Kenneth Jones and Tema Okun, ChangeWork, 2001, available at http://www.cwsworkshop.org/PARC_site_B/dr-culture.html

6 See for example: Know Thyself: How to Write a Reflexivity Statement, by Sherry Hammy Ph.D., Psychology Today, May 22, 2018, available at https://www.psychologytoday.com/us/blog/the-web-violence/201805/know-thyself-how-write-reflexivity-statement
